# Development of an insecticidal nanoemulsion with *Manilkara subsericea* (Sapotaceae) extract

**DOI:** 10.1186/1477-3155-12-22

**Published:** 2014-05-18

**Authors:** Caio Pinho Fernandes, Fernanda Borges de Almeida, Amanda Nunes Silveira, Marcelo Salabert Gonzalez, Cicero Brasileiro Mello, Denise Feder, Raul Apolinário, Marcelo Guerra Santos, José Carlos Tavares Carvalho, Luis Armando Cândido Tietbohl, Leandro Rocha, Deborah Quintanilha Falcão

**Affiliations:** 1 Programa de Pós, Graduação em Biotecnologia Vegetal, Centro de Ciências da Saúde, Universidade Federal do Rio de Janeiro – UFRJ, Bloco K, 2° andar – sala 032, Av. Brigadeiro Trompowski s/n, CEP: 21941-590 Ilha do Fundão, RJ, Brazil; 2 Laboratório de Farmacotécnica, Colegiado de Ciências Farmacêuticas, Universidade Federal do Amapá, Campus Universitário Marco Zero do Equador, Rodovia Juscelino Kubitschek – KM – 02-Jardim Marco Zero, CEP: 68903-419 Macapá, AP, Brazil; 3 Laboratório de Tecnologia Farmacêutica I, Faculdade de Farmácia, Universidade Federal Fluminense, Rua: Mario Viana, 523, Santa Rosa, CEP: 24241-000 Niterói RJ, Brazil; 4 Laboratório de Biologia de Insetos – LABI, Departamento de Biologia Geral (GBG), Universidade Federal Fluminense, Morro do Valonguinho S/No, CEP 24001-970 Niterói, RJ, Brazil; 5 Faculdade de Formação de Professores, UERJ, Rua: Dr. Francisco Portela, 1470 – Patronato, CEP: 24435-005 São Gonçalo, Rio de Janeiro, Brazil; 6 Laboratório de Pesquisa em Fármacos, Colegiado de Ciências Farmacêuticas, Universidade Federal do Amapá, Rodovia Juscelino Kubitschek – KM – 02 – Jardim Marco Zero, CEP: 68903-419 Macapá, AP, Brazil; 7 Laboratório de Tecnologia de Produtos Naturais – LTPN, Departamento e Tecnologia Farmacêutica, Faculdade de Farmácia, Universidade Federal Fluminense – UFF Rua, Mario Viana, 523, CEP: 24241-000, Santa Rosa, Niterói, RJ, Brazil

**Keywords:** *Dysdercus peruvianus*, *Manilkara subsericea*, Nanoemulsions

## Abstract

**Background:**

Plants have been recognized as a good source of insecticidal agents, since they are able to produce their own defensives to insect attack. Moreover, there is a growing concern worldwide to develop pesticides with low impact to environment and non-target organisms. Hexane-soluble fraction from ethanolic crude extract from fruits of *Manilkara subsericea* and its triterpenes were considered active against a cotton pest (*Dysdercus peruvianus*). Several natural products with insecticidal activity have poor water solubility, including triterpenes, and nanotechnology has emerged as a good alternative to solve this main problem. On this context, the aim of the present study was to develop an insecticidal nanoemulsion containing apolar fraction from fruits of *Manilkara subsericea.*

**Results:**

It was obtained a formulation constituted by 5% of oil (octyldodecyl myristate), 5% of surfactants (sorbitan monooleate/polysorbate 80), 5% of apolar fraction from *M.* subsericea and 85% of water. Analysis of mean droplet diameter (155.2 ± 3.8 nm) confirmed this formulation as a nanoemulsion. It was able to induce mortality in *D. peruvianus*. It was observed no effect against acetylcholinesterase or mortality in mice induced by the formulation, suggesting the safety of this nanoemulsion for non-target organisms.

**Conclusions:**

The present study suggests that the obtained O/A nanoemulsion may be useful to enhance water solubility of poor water soluble natural products with insecticidal activity, including the hexane-soluble fraction from ethanolic crude extract from fruits of *Manilkara subsericea*.

## Background

Chemical pesticides have been used to control pest insects, however, they are usually toxic to environment. There is a growing concern worldwide regarding indiscriminate use of these substances, which are associated to environmental pollution and toxicity risk to non-targeted organisms [1]. Plant species are well recognized by their ability to produce defensive substances, in order to protect themselves from insect attack [2]. These natural products appear as potential sources of new biodegradable insecticides with wide range of mechanisms of action, being an important alternative for insect pest management in agriculture [3]. One of the most promising and recognized group of substances with insecticidal activity are the triterpenes [4].

*Manilkara subscericea* (Mart.) Dubard (Sapotaceae) is an endemic species of Brazilian Atlantic Forest [5] and widely distributed at Restinga de Jurubatiba National Park (Rio de Janeiro State, Brazil) [6]. Several non-polar pentacyclic triterpenes have been described as major constituents of *M. subsericea*, mainly alpha- and beta-amyrin esters [7,8]. Hexane-soluble fraction from ethanolic crude extract from fruits of *M. subsericea* and its major substances (alpha- and beta-amyrin acetate) was able to induce mortality, delayed development and inhibition of moulting in *Dysdercus peruvianus *[9], a hemiptera species which causes serious loss of cotton crops [10]. This apolar fraction and its triterpenes have poor water solubility and are soluble in toxic organic solvents, such as chloroform and dichloromethane, being this intrinsic characteristic a technological challenge if development if a viable product is desired.

Nanotechnology has emerged as a promising area for development of products in a wide range of applications, including pesticide agents. Considering that many of the insecticides known today are organic compounds with poor water solubility, development of nanoproducts appear to solve this main problem, enhancing water solubility, bioavailability and resulting in stable formulations without utilization of organic toxic solvents [11]. Nanoemulsions are one of the most important formulations to enhance solubility and dissolution properties of poorly water soluble substances [12]. They are also referred as miniemulsions or ultrafine emulsions and have small droplet size (20-200 nm). They are transparent or translucent, often presenting a bluish reflect and have high kinetic stability [13]. Low energy methods have been used to achieve nanoemulsions, including reverse-phase composition (RPC) and temperature of inversion phase (TIF) [14]. Formulation screening stage is crucial if development of a stable nanoformulation is desired, especially if a low energy method is employed, being determination of required HLB value of an oil [15] and construction of pseudo-ternary phase diagrams [16] very useful, especially to achieve nanoemulsions.

On this context, the aim of the present study was to develop an insecticidal nanoemulsion containing apolar fraction from fruits of *Manilkara subsericea* and verify its effects against *Dysdercus peruvianus* and non-target organisms.

## Results and discussion

Preliminary solubility studies were performed regarding choice of oil phase and surfactants. Octyldodecyl myristate (MOD®) was the best oil, being able to solubilize equal amount (1:1, w/w) of hexane-soluble fraction from fruits of *M. subsericea* (HF). It is frequently necessary to use blends, such as a pair of hydrophilic and lipophilic non-ionic surfactants, to achieve droplets with small diameter [17]. Sorbitan oleate and polysorbate 80 were considered the best pair (Data not shown). These surfactants have been used in low energy methods, being able to produce nanoemulsions with smaller mean droplet size, when compared to other surfactants. This could be explained by the ability of this couple to induce formation of a looser film, which is associated to generation of nanoemulsions [12]. Addition of water to a surfactant in oil solution was employed in the present study, since it provided better results, when compared to addition of oil to an aqueous surfactant solution (Data not shown). This could be attributed to phase transitions and changes in the curvature of the surfactant from W/O to O/W during emulsification process [18].

In order to predict the best ratio of surfactants to be used, several emulsions were prepared varying the relative amounts of sorbitan oleate and polysorbate 80. Most of them presented instable behavior, including critical macroscopical changes, such as creaming and phase separation. Surfactants can be classified according to their Hydrophile-Lipophile Balance (HLB), a semi-empirical scale [19] and several HLB values can be obtained using different amounts of each component of a couple of surfactants [20]. Emulsions with HLB values of 10 (sorbitan oleate/polysorbate ratio, 1.0/1.1) and 11 (sorbitan oleate/polysorbate ratio, 1.0/1.7) were considered more stable. A second set of emulsions within this HLB range was prepared and the obtained formulations presented translucent aspect and bluish reflect, which is characteristic for nanoemulsions [13]. Mean droplet size analysis indicated that nanoemulsion with HLB value of 10.75 (sorbitan oleate/polysorbate ratio, 1.0/1.5) presented the smallest mean diameter (50.6 ± 0.4 nm) and low polydispersity (0.164 ± 0.021). Stable formulations with low mean droplet size can be obtained when HLB value of the surfactant couple coincides with required HLB value of the oil [12,20]. Thus, required HLB of oil can be determined by calculating the HLB value of emulsifier or emulsifier mixture which was able to induce formation of the most stable formulation, among a set of emulsions prepared with different blends of a couple of emulsifiers in a wide range of HLB value [21]. Our results indicate that 10.75 should be the required HLB value of MOD® used in the present study.

**Figure 1:**
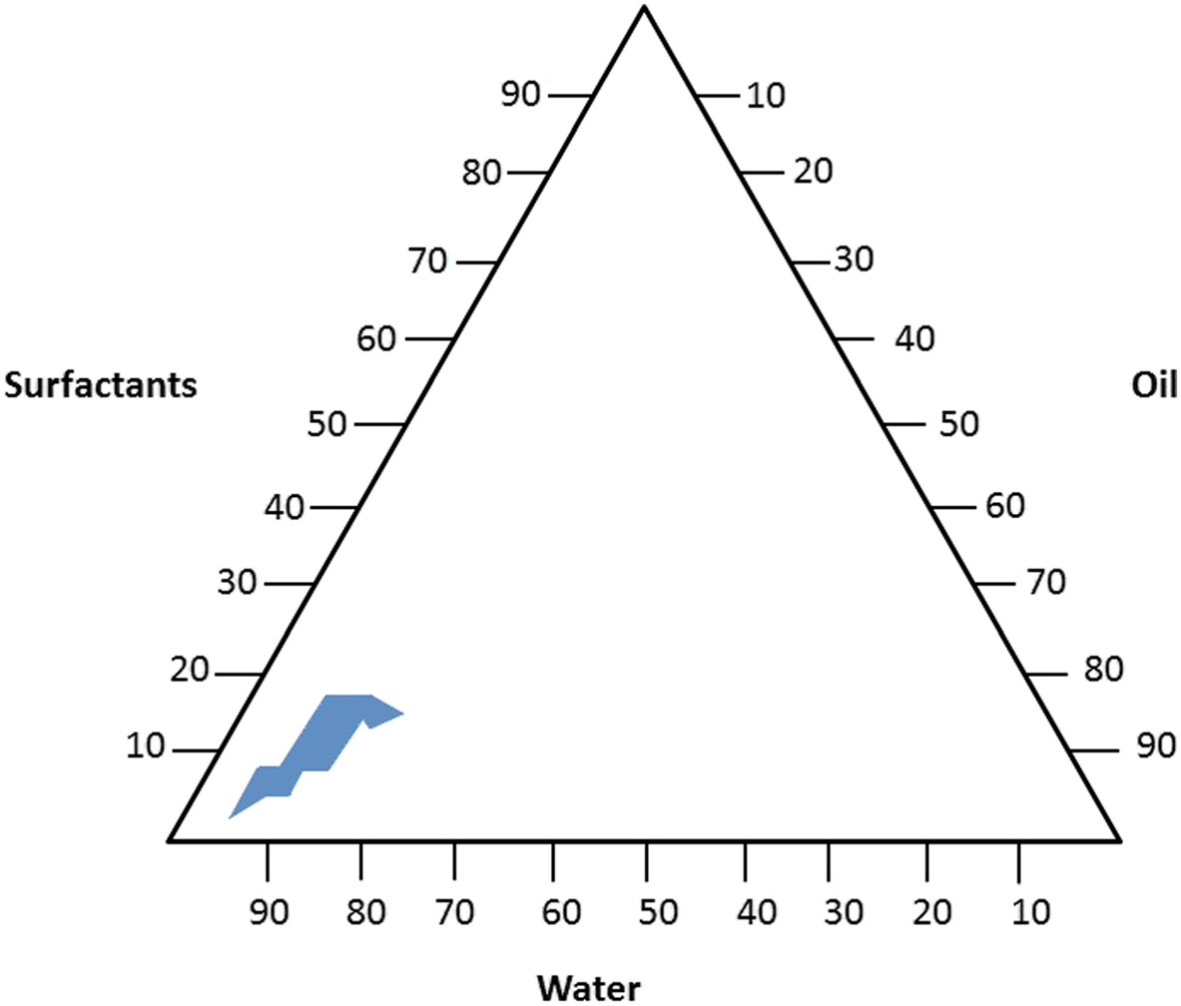
**Pseudo-ternary phase diagram constructed with water, MOD® and surfactants (sorbitan monoleate/polysorbate 80, HLB =10.75) at different compositions.** Nanoemulsion region is delimited in blue.

It is observed that not every combination of components produces nanoemulsions over the whole range of possible compositions [22]. Thus, a total of 28 emulsions were prepared using different percentages of water, MOD® and surfactants (sorbitan monoleate/polysorbate 80, HLB 10.75) and mean droplet size of each formulation was analyzed. Mean droplet size ranged from 45.9 ± 0.4 nm (oil 15%, surfactants 15%, water 70%) to 421.5 ± 50.4 nm (oil 2.5%, surfactants 7.5%, water 90%) (Table 1). Composition of each emulsion obtained can be expressed as a pseudo-ternary phase diagram, which is represented by equilateral triangle in which four or more constituents are investigated [22] and is very useful to determine relation between phase behavior of a mixture and its composition [23]. In all, 23 emulsion presented mean droplet size bellow 200 nm, ranging 45.9 ± 0.4 nm to 196.4 ± 12.5 and were used to delineate the nanoemulsion region (Figure 1). Small mean droplet sizes, such as 48.7 ± 0.2 nm (7.5% of oil, 10% of surfactants, 82.5% of water), 51.7 ± 0.2 nm (10% of oil, 12.5% of surfactants, 77.5% of water) and 45.9 ± 0.4 nm (15% of oil, 15% of surfactants, 70% of water) were obtained (Table 1). This data may be important for further studies or development of nanoformulations using MOD®, sorbitan oleate, polysorbate 80 and water.

**Table 1:** Composition, mean droplet size and polydispersity of each formulation prepared during construction of pseudo-ternary phase diagram for delimitation of nanoemulsion region

	**% of oil**	**% of surfactants**	**% of water**	**Mean diameter (nm)**	**Polydispersity**
1^a^	5	5	90	50.6 ± 0.4	0.164 ± 0.021
2	2.5	5	92.5	234.2 ± 12.5	0.025 ± 0.012
3	2.5	7.5	90	421.5 ± 50.4	0.005 ± 0.000
4^a^	5	7.5	87.5	196.4 ± 12.5	0.178 ± 0.044
5^a^	7.5	5	87.5	145.7 ± 8.6	0.132 ± 0.038
6	7.5	2.5	90	256.9 ± 8.6	0.016 ± 0.011
7^a^	5	2.5	92.5	151.7 ± 6.0	0.155 ± 0.018
8^a^	10	5	85	139.4 ± 9.6	0.078 ± 0.049
9^a^	10	7.5	82.5	133.5 ± 0.5	0.247 ± 0.011
10^a^	7.5	7.5	85	139.5 ± 4.7	0.073 ± 0.039
11^a^	10	10	80	86.8 ± 0.9	0.294 ± 0.006
12^a^	7.5	10	82.5	48.7 ± 0.2	0.313 ± 0.002
13	5	10	85	234.4 ± 2.3	0.270 ± 0.016
14	5	12.5	82.5	298.5 ± 31.8	0.005 ± 0.000
15^a^	7.5	12.5	80	85.4 ± 1.2	0.350 ± 0.005
16^a^	10	12.5	77.5	51.7 ± 0.2	0.331 ± 0.005
17^a^	7.5	15	77.5	162.4 ± 1.3	0.335 ± 0.011
18^a^	7.5	17.5	75	159.2 ± 2.3	0.334 ± 0.007
19^a^	10	15	75	68.7 ± 0.8	0.360 ± 0.004
20^a^	10	17.5	72.5	87.9 ± 1.5	0.365 ± 0.003
21^a^	12.5	15	72.5	170.6 ± 6.2	0.144 ± 0.027
22^a^	12.5	12.5	75	66.6 ± 1.1	0.304 ± 0.005
23^a^	12.5	10	77.5	120.1 ± 1.0	0.225 ± 0.003
24^a^	15.0	12.5	72.5	95.3 ± 0.6	0.255 ± 0.006
25^a^	15	15	70	45.9 ± 0.4	0.271 ± 0.005
26^a^	12.5	7.5	80	75.4 ± 2.3	0.340 ± 0.005
27^a^	17.5	15.0	67.5	161.6 ± 1.9	0.266 ± 0.007
28^a^	12.5	17.5	70	97.2 ± 1.0	0.256 ± 0.002

Oil – MOD®.

Surfactants – sorbitan monooleate/polysorbate 80 at HLB of 10.75.

^a^Formulations in the nanoemulsion region.

Special attention was given to formulation comprised by 5% of MOD, 5% of surfactants and 90% of water, which also presented stable behavior, small mean droplet size (50.6 ± 0.4 nm) and low polidispersity (0.164 ± 0.021). Low surfactant percentage could be considered an advantage, since further preparation of this formulation would reduce toxicity and costs with raw materials, when compared to other nanoemulsions with higher concentrations of surfactants. Thus, this formulation was chosen to prepare a nanoemulsion with hexane-soluble fraction from fruits of *Manilkara subsericea* dispersed through internal phase (HFNE).

**Figure 2:**
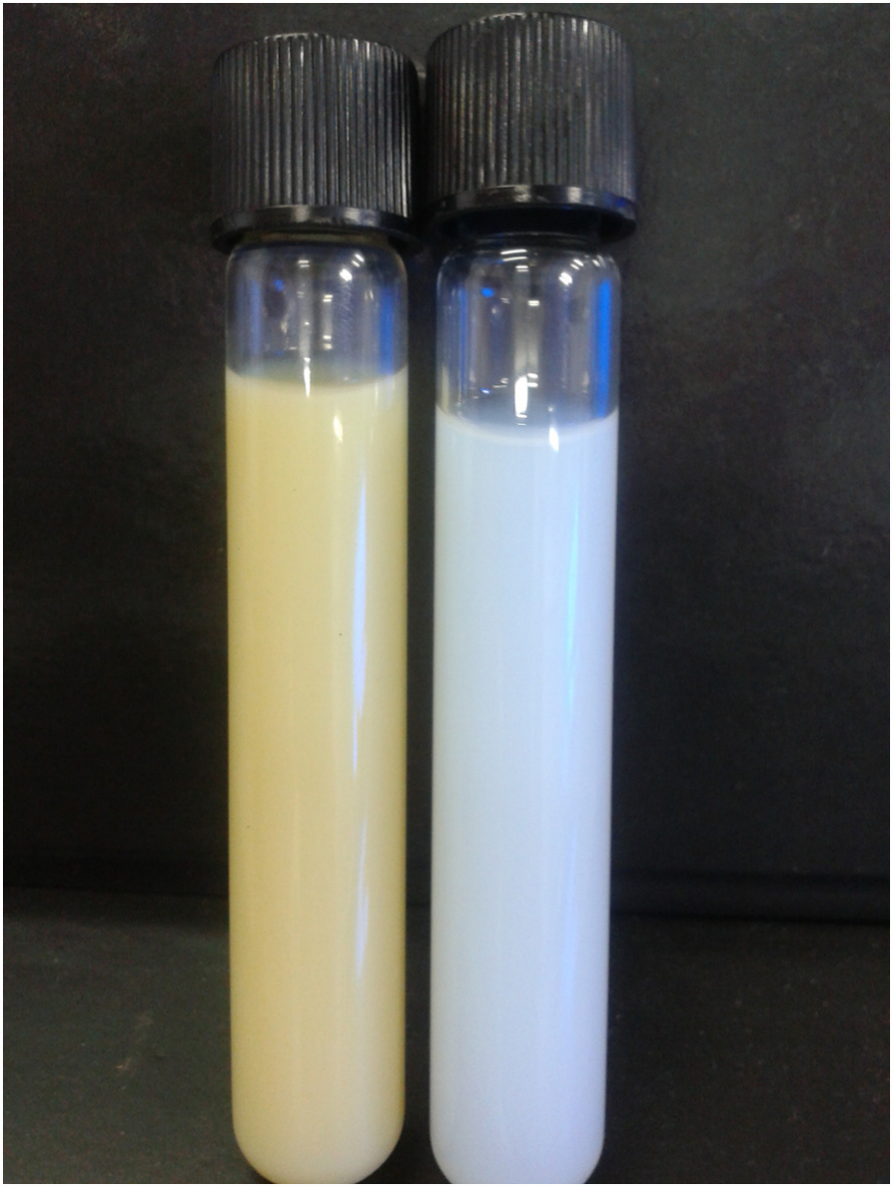
**Nanoemulsions obtained by low energy method.** HFNE shown in left side and blank nanoemulsion shown in right side of the picture.

**Figure 3:**
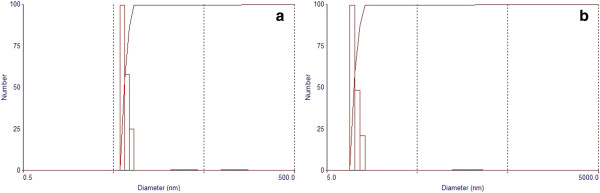
**Particle size distribution of (a) negative control (57.0 ± 0.3 nm) and (b) nanoemulsion with hexane-soluble fraction from fruits of *M. subsericea *(155.2 ± 3.8 nm).** Polidispersity was 0.270 ± 0.006 for blank nanoemulsion and 0.150 ± 0.050 for nanoemulsion with hexane-soluble fraction from fruits of *M. subsericea*.

Concentration of extract corresponded to equal percentage of MOD®, based on its intrinsic solubility. This amount was discounted from water percentage, being HFNE constituted by 5% of MOD, 5% of surfactants, 5% of hexane-soluble fraction from fruits of *M. subsericea* and 85% of water. A blank nanoemulsion without hexane-soluble fraction of *M. subsericea* extract (HF) was prepared for negative control. Both nanoemulsions presented a characteristic bluish reflect, associated to Tyndall effect [13] (Figure 2). It was observed an increase in the nanoemulsion mean droplet size when HF was dispersed through oil phase (Figure 3). This fact could be explained due to deposition of substances, which may reduce the flexibility of the surfactant film and result in more compact films instead of looser films and smaller mean droplets [12]. Previous gas chromatography analysis of HF indicated a high relative percentage of pentacyclic triterpenes, including beta-amyrin acetate (10.27%), alpha-amyrin acetate (42.34%), beta-amyrin caproate (5.46%), alpha-amyrin caproate (7.26%), beta-amyrin caprylate (2.44%) and alpha-amyrin caprylate (5.04%) [8]. These substances may be contributing to the result described above.

HFNE and blank nanoemulsion presented zeta potential values of – 47.4 ± 3.2 and – 59.6 ± 4.1, respectively. Zeta potential is a special parameter that should be analyzed, in order to determine stability of nanoemulsions and is associated to surface potential of the droplets [24]. Maximum stability is observed when zeta potential value is above ± 30 mV [25]. The high stability of formulations with great zeta potential values is associated to repulsive forces that exceed attracting Van der Waals forces, resulting in dispersed particles and a deflocculated system [23]. Macroscopical analysis of the nanoemulsion with HF and blank nanoemulsion indicated that these formulations maintained their original fine appearance and bluish reflection. It was observed no phase separation, creaming and sedimentation under room temperature (25 ± 2°C) and accelerated stability evaluation. Long term physical stability of a nanoemulsion related to its small droplets, making this type of formulation being also referred as “approaching thermodynamic stability” [26,13].

**Figure 4:**
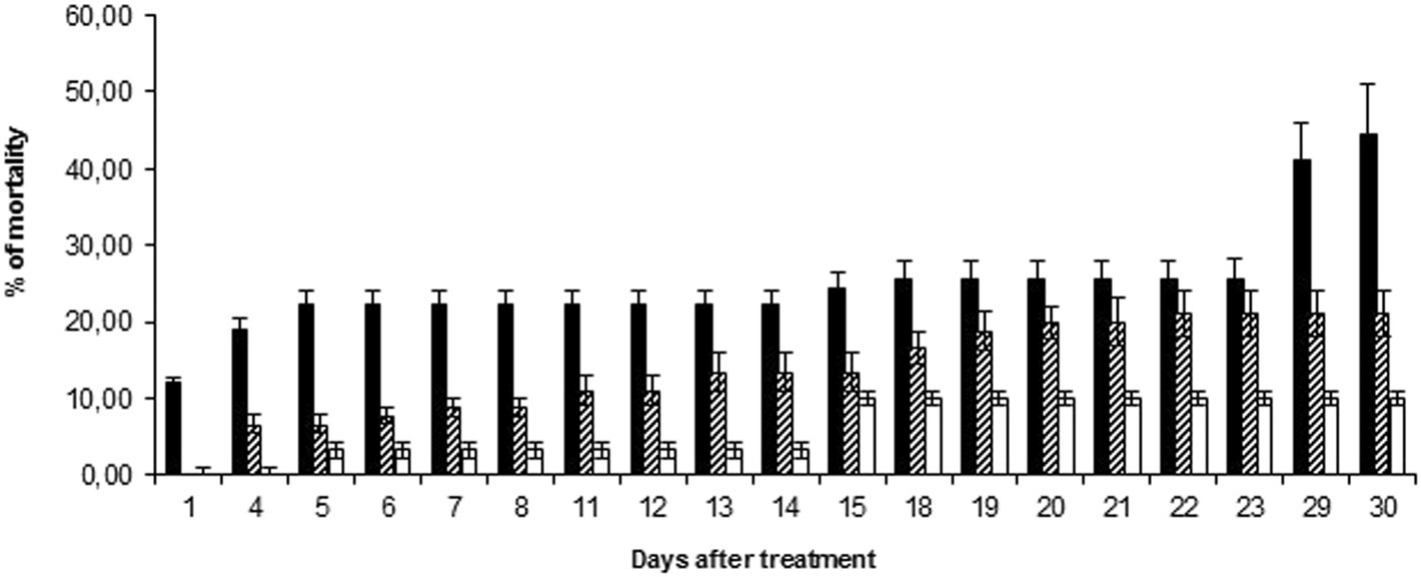
**Analysis of mortality after topical treatment of *Dysdercus peruvianus *with nanoemulsion containing hexane-soluble fraction from fruits of *Manilkara subsericea *(HFNE) (filled column).** Negative control group was topically applied with blank nanoemulsion (crosshatched columns). Untreated group is represented by open columns. Each group represents mean of three experiments.

Insecticidal assay was performed in order to verify if HFNE is able to induce mortality in *D. peruvianus*. During the whole experimental period, it was observed that HFNE (treated group) did not interfere in body weight, when compared to untreated group, indicating the absence of antifeedant effect. This effect was also not detected in negative control group. It was not observed overaged, extranumerary nymphs or insects with body deformations. Figure 4 indicates that mortality in the untreated group ranged from 3.3 ± 1.15%, between 5° and 14° days of observation, to 10 ± 1.53%, between 15° and 30° of observation. Negative control group (treated with blank nanoemulsion) presented higher levels of mortality throughout the experimental period, reaching (6.6 ± 1.15%) (p < 0.001) after 4 days, 13.3 ± 2.52% (p < 0.001) after 14 days and 21.10 ± 3.06% (p < 0.001) after 30 days of treatment. Treatment of insects with HFNE exhibited significantly higher levels of mortality. It was observed that mortality began on the first day after treatment (12.23 ± 0.58%) (p < 0.001), reached 22.23 ± 1.73% (p < 0.001) after 14 days and 44.43 ± 6.66% (p < 0.0001) after the end of the experiment. Significant differences between the group of insects treated with hexanic nanoemulsion containing extract of *M. subsericea* and the control group were detected in almost all days of observation until the end of the experiment (ANOVA, p < 0.005). However, it is worth to note that there was no statistical difference between HFNE-treated and blank nanoemulsion-treated insects among days 21-23 after treatment. Perhaps, differences in the speed of absorption between HFNE and blank nanoemulsion by insect metabolic systems may explain this not expectable result. Physiological mechanisms of metabolization of these compounds by invertebrates remains unknown [9] and is now under investigation by our research group.

Changes in the time period in which occur the processes of molt and metamorphosis were observed in group treated with HFNE and negative group (Data not shown). Moreover, an associated high mortality rate were displayed continuously and gradually increasing throughout insects lifecycle regardless whether the insects were in the nymphal or adult stage. This observation point out to a physiological connection between the neuroendocrine control of the insect development and the reduced longevity obtained after treatments. These results suggest that HFNE may able to release insecticidal components from HF, while formulation used as blank nanoemulsion may be used to disperse other insecticidal agents.

**Figure 5:**
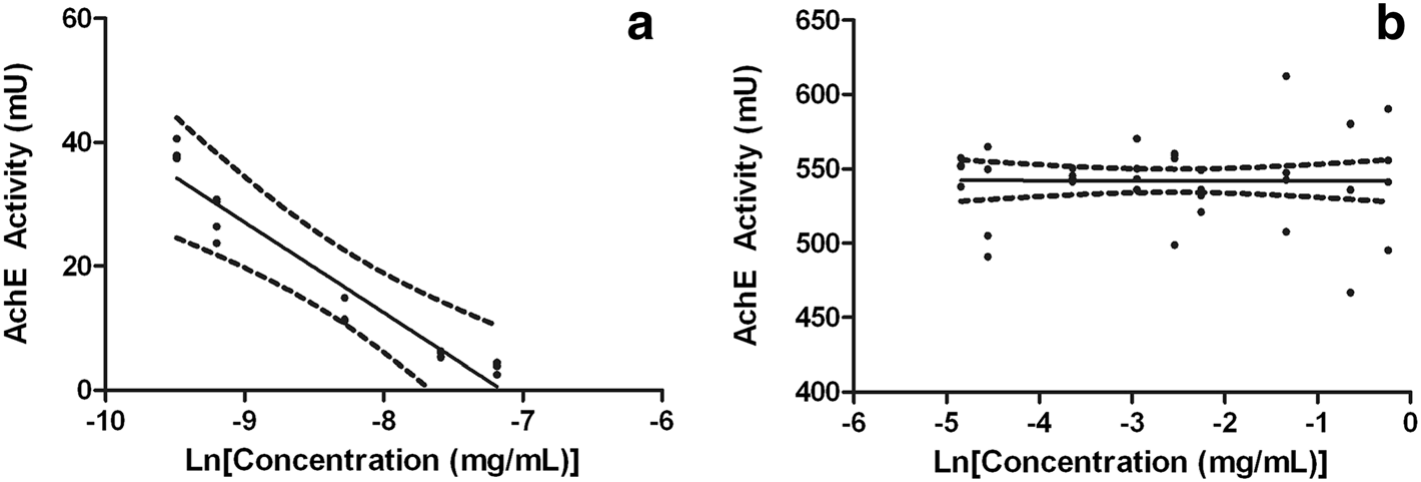
**Linear regression between AchE activity (mU) x natural logarithm of (a) effective concentration of eserine (p < 0.05) and (b) effective concentration of hexane-soluble fraction from fruits of *Manilkara subsericea *** **(p > 0.05).**

In order to evaluate if the formulation interfere with acetylcholinesterase, HFNE was tested using a colorimetric assay. Positive control was performed by preparing a nanoemulsion with eserine, a recognized lipophilic anticholinesterase agent dispersed through oil phase (MOD®). Mean droplet analysis confirmed this formulation, constituted by 5% of MOD®, 5% of surfactants (HLB of 10.75), 0.05% of eserine and 89.95% of water, as a nanoemulsion (67.3 ± 0.3 nm). IC50 of this substance could not be determined, since lowest eserine concentration (0.6 ppm) was able to inhibit 90% of enzyme activity (Figure 5). Results suggest that eserine may be able to displace from disperse phase of the nanoemulsion to the aqueous external phase and induced a dose-dependent inhibition of acetylcholinesterase, since it should be in external phase to bind to the enzyme. Pesticides are used as an important tool to protect crops worldwide, however, residues of these substances can be found in many environments, including rivers, estuaries and oceans [27,28]. Most insecticides provide harmful impacts on non-target species, especially aquatic organisms, such as fishes. These animals are especially susceptive to acetylcholinesterase inhibitors, probably due to lacking of detoxification systems and sharing same neurological and respiratory mechanisms [27,29]. Acetylcholinesterase inhibitory assay indicated that no significant inhibition of acetylcholinesterase modulated by HFNE (Figure 5). Considering that acetylcholinesterase used in this assay is from fish origin, our results suggest that HFNE may not induce harmful effects over aquatic non-target animals, indicating the potential of this nanoemulsion as an insecticidal agent. Nanoemulsion without *M. subsericea* extract also did not interfere with the enzyme (Data not shown).

Acute toxicity evaluation was performed in order to verify effects of HFNE in mice. It was not observed any behavioral change during all tested period and mortality in all groups. Analysis of body weight also indicated absence of significant difference between HFNE and negative control group (Table 2). It was also not observed significant difference in food and water consumption, macroscopical aspects and weight of organs between groups treated with HFNE and negative control group (Data not shown). Treatment with HFNE was performed at a single high dose corresponding to 3 g/kg of extract per animal. Since no death or toxic signals were observed, LD50 could not be estimated and HFNE, suggesting that HFNE may be considered non-toxic [30].

**Table 2:** **Weight variation in adult female and male Swiss albino mice (*Mus musculus *) treated with HFNE (5% of MOD®, 5% of surfactants (HLB of 10.75), 5% of hexane-soluble fraction from fruits of *M. subsericea *and 85% of water) by oral route, corresponding to 3 g/kg of extract**

	**Body weight (Male)**		**Body weight (Female)**	
	**Initial (g)**	**Final (g)**	**Initial (g)**	**Final (g)**
HFNE	49.06 ± 0.43	50.39 ± 1.37	52.72 ± 1.62	50.64 ± 0.63
Control	50.65 ± 1.50	52.05 ± 2.71	50.64 ± 0.63	51.76 ± 1.59

Control groups received same volume of blank nanoemulsion (5% of MOD, 5% of surfactants and 90% of water).

p > 0.05.

## Conclusions

Previous study performed by our research group indicated that hexane-soluble fraction from ethanolic crude extract from fruits of *Manilkara subsericea* presented insecticidal activity against *Dysdercus peruvianus*. This activity may be partially attributed to beta-and alpha amyrin acetates, which may be used as chemical markers for quality control of products with *M. subsericea* extracts. However, these substances, as well as the active fraction are poorly water soluble. As part of our ongoing studies with this species, we decided to develop an insecticidal nanoemulsion. This formulation was able to induce mortality in insects and our results suggest that it may be safe for non-target organisms and environment. The present study suggests the obtained O/A nanoemulsion may be useful to enhance water solubility of poor water soluble natural products with insecticidal activity, including the hexane-soluble fraction from ethanolic crude extract from fruits of *Manilkara subsericea*. The absence of organic toxic solvents and stability makes this nanoemulsion a potential insecticidal product.

## Materials and methods

### Chemicals

Sorbitan oleate (HLB: 4.3) and Polysorbate 80 (HLB: 15) were purchased from La Belle Ativos Ltda (Paraná, Brazil). Octyldodecyl myristate (MOD®) was purchase from Brasquim Ltda (São Paulo, Brazil). Acetylthiocholine iodide (ATCI), 5,5-dithiobis-2-nitrobenzoic acid (DTNB), physostigmine (eserine) and acetylcholinesterase from electric eel (type VI-S, C3389-2UK, lyophilized powder) were purchased from Sigma (Sigma-Aldrich Corporation, St Louis, MO). Hexane-soluble fraction from fruits of *M. subsericea* was previously obtained [9] and stored at 4°C for further utilization.

### Emulsification method

Emulsions were prepared by temperature of inversion phase method [31]. The required amounts of both emulsifiers were dissolved in the oil phase and heated at 75 ± 5°C, while the aqueous phase was separately heated at same temperature. When both phases reached the same temperature, aqueous phase was gently added and mixed with the oil phase, using a mechanic agitator model Fisatom 713D at 400 rpm for 10 min and additional 5 min of agitation under cooling. Aditional constituents was weight an placed together with oil and surfactants mixture, being its mass discounted from water mass.

### Required HLB determination

Each emulsion was prepared at a final mass of 25 g, containing 90% (w/w) of distilled water, 5% (w/w) of MOD® and 5% of a mixture of emulsifiers [32]. Series of emulsions were prepared using sorbitan oleate (HLB = 4.3) and polysorbate 80 (HLB = 15), allowing a wide range of HLB values from 4.3 (5% w/w of sorbitan oleate) to 15 (5% w/w of polysorbate 80) by blending together the emulsifiers in different ratios.

### Pseudo-ternary phase diagram

Nanoemulsion region was determined using pseudo-ternary phase diagram. Each corner corresponded to 100% of water, surfactants and MOD®. Surfactants blend was kept constant and corresponded to ratio which results on required HLB value of oil phase. Composition (w/w) which allowed required HLB value determination was used as starting point (90% of distilled water, 5% of oil and 5% of surfactants blend) and mean droplet size of each prepared composition was performed in order to determine nanoemulsion region.

### Macroscopical analysis

Stability of all emulsions was evaluated immediately and after 1, 15 and 30 days of manipulation by macroscopic analysis, such as color, visual aspect, phase separation, creaming and sedimentation. During this period all emulsions were maintained under room temperature (25 ± 2°C) in screw-capped glass test tubes [32]. Acelerated stability evaluation was performed keeping emulsion under controlled temperature (40 ± 5°C).

### Droplet size and zeta potential analysis

The droplet size, polydispersity **and zeta potential** were determined by photon correlation spectroscopy using a ZetaPlus (Brookhaven Inst. Corp., USA). Each emulsion was diluted using ultra-pure Milli-Q water (1:25). Measurements were performed in quintuplicate and average droplet size was expressed as the mean diameter.

### Insect bioassay

*Dysdercus peruvianus* were obtained from the colony maintained in the Laboratory of Insect Biology of the Universidade Federal Fluminense (GBG-UFF), being kept at 24-25°C, relative humidity of 70-75% and a 16:8 h light:dark cycle [9].

Fourth-instar insects were randomly chosen and separated in two treated groups, being one group topically applied with a nanoemulsion containing hexane-soluble fraction from fruits of *M. subsericea* (HFNE) (5% of MOD®, 5% of surfactants (HLB of 10.75), 5% of HF and 85% of water), corresponding to 50 μg of extract per insect, while negative control group was treated with blank nanoemulsion (5% of MOD®, 5% of surfactants and 90% of water). Untreated insects received no treatment, being only fed. Biological evaluation was performed in order to determine mortality levels during the entire time required for development from the fourth instar to the adult stage [9,33,34]. All experiments were repeated at least three times with samples from 30 insects (*n* = 30 in each triplicate). Significance of the results was analysed using ANOVA and Tukey’s test21 according to Stats Direct Statistical Software, v.2.2.7 for Windows 98. Differences between treated group and control.

### Anticholinesterase assay

Anticholinesterase activity was performed according to method described by Ellman et al. (1961) [35] with some modifications [36], using a 96-well microplate. A total volume of 200 μL of test media was composed by 65 μL of Phosphate buffered saline (PBS), 60 μL of 5,5′-dithiobis-(2-nitrobenzoic acid) (DTNB) 1,5 mM, 25 μL of electric eel acetylcholinesterase (Sigma) (AchE) 550 mU/mL, 25 μL of nanoemulsion and 25 μL of acetylthiocholine iodide (ASCh). Different concentrations of HF and eserine (positive control) were obtained by dilution of each nanoemulsion with PBS. Negative control was performed using a blank nanoemulsion, without inhibitor or extract. The spontaneous hydrolysis of substrate was calculated replacing the enzyme solution by PBS. Absorbance was measured at 412 nm. The statistical analysis of the anticholinesterase assay was performed on GraphPad Prism 5.04 program using Pearson’s correlation coefficient with 95% confidence interval.

### Acute toxicity

#### ***Animals***

This study was approved by the Ethics Committee of the Universidade Federal do Amapá (CEP – UNIFAP – 005AP/2013). All procedures were performed according to the International Committee for animal care in accordance with established national regulations for animal experimentation. The experiments were performed using adult female and male Swiss albino mice (*Mus musculus*), 12 weeks age, provided by the Central Laboratory of the State of Amapá – Macapá (LACEN/AP). Each experimental group was composed of 5 animals. They were kept in polyethylene cages on a temperature-controlled rack (25°C ± 2°C) under a 12-hour light-dark cycle. They had free access to food and water, except for the 24 hours before the experiments, when they had access only to water.

#### ***Experimental protocol***

Acute toxicity studies were performed using both sexes of mice according to Pina et al. (2012) [30], with some modifications. Treated groups received a single dose of HFNE (5% of MOD®, 5% of surfactants (HLB of 10.75), 5% of hexane-soluble fraction from fruits of *M. subsericea* and 85% of water) by oral route, corresponding to 3 g/kg of extract. Negative control groups received a blank nanoemulsion (5% of MOD®, 5% of surfactants and 90% of water).

Observations were performed at 30, 60, 120, 240, 360 and 720 min after the oral treatment and daily for fourteen days. Behavioral changes (agitation, convulsions, vocal fremitus, irritation, stereotyped movements, touch response, salivation, tremors, writhing, body distension, ptose, sleepiness, defecation, diarrhea, piloerection), weight, food and water intake, clinical signs of toxicity and mortality were recorded daily. At the end of fourteen days, they were sacrificed by cervical dislocation and taken to autopsy for macroscopic observation of the organs (heart, lung, liver, kidney and spleen). Statistical analysis was performed by Student t test with 95% confidence intererval, using GraphPad Prism 5.04. Differences between organs, body weight and food and water intake were considered significant when p < 0.05.

## Competing interests

All authors declare no conflict of interests.

## Authors’ contributions

CPF contributed in collecting plant sample, running the laboratory work, analysis of the data and drafted the paper. FBA and ANS contributed in preparation of extracts, HLB determination and nanoemulsions preparation. MSG, CBM and DF contributed in insect bioassay. MGS contributed in plant identification and herbarium confection. LACT contributed in AChE bioassay. JCTC contributed to critical reading of the manuscript and acute toxicity assay. LR and DQF designed the study, supervised the laboratory work and contributed to critical reading of the manuscript. All the authors have read the final manuscript and approved the submission.

## Authors’ information

Caio Pinho Fernandes is a professor at Universidade Federal do Amapá and has been working with natural products, including phytochemistry, nanotechnology and biological activities of these compounds.

Fernanda Borges de Almeida is an undergraduate student at Universidade Federal do Amapá and participated in this project as part of her scientific initiaion program.

Amanda Nunes Silveira is an undergraduate student at Universidade Federal Fluminense and participated in this Project as part of her scientific initiaion program.

Marcelo Salabert Gonzalez is professor at Universidade Federal Fluminense and has been working with complementary strategies to control insects with secondary metabolites from plant species.

Cicero Brasileiro Mello is professor at Universidade Federal Fluminense and has been working with complementary strategies to control insects with secondary metabolites from plant species.

Denise Feder is professor at Universidade Federal Fluminense and has been working with complementary strategies to control insects with secondary metabolites from plant species.

Raul Apolinário is undergraduate student at Universidade Federal Fluminense and participated in this project as part of her scientific initiation program and did al experiments with insects.

Marcelo Guerra Santos is professor at Universidade Estadual do Rio de Janeiro. He is a botanist and has been working with species from sandbanks of Parque Nacional da Restinga de Jurubatiba (RJ) Brazil.

José Carlos Tavares Carvalho is professor and President of the Universidade Federal do Amapá (Brazil) and has been working with natural products pharmacology.

Luis Armando Cândido Tietbohl is a Master’s student at Universidade Federal Fluminense and has been working with acetylcholinesterase inhibition.

Leandro Rocha is professor at Universidade Federal Fluminense and has been working with natural products and its biological activities.

Deborah Quintanilha Falcão is professor at Universidade Federal Fluminense and has been working with nanotechnology of natural products.
